# Carboxylesterase-Cleavable Biotinylated Nanoparticle for Tumor-Dual Targeted Imaging

**DOI:** 10.7150/thno.37625

**Published:** 2019-09-25

**Authors:** Peiyao Chen, Wen Kuang, Zhen Zheng, Shuye Yang, Yaling Liu, Lanhong Su, Kui Zhao, Gaolin Liang

**Affiliations:** 1 Hefei National Laboratory of Physical Sciences at Microscale, Department of Chemistry, University of Science and Technology of China, 96 Jinzhai Road, Hefei, Anhui 230026, China; 2 Department of PET Center, The First Affiliated Hospital, College of Medicine, Zhejiang University, 79 Qingchun Road, Hangzhou, Zhejiang 310003, China; 3 Jiangsu Institute of Nuclear Medicine, 20 Qianrong Road, Wuxi, Jiangsu 214063, China; 4 School of Life Sciences, University of Science and Technology of China, 443 Huangshan Road, Hefei, Anhui 230027, China

**Keywords:** near-infrared, nanoparticle, carboxylesterase, biotin receptor, tumor imaging

## Abstract

Near-infrared (NIR) nanoprobes with fluorescence “Turn-On” property are advantageous in cancer diagnosis but, to the best of our knowledge, “smart” nanoprobe that simultaneously targets both biotin receptor and carboxylesterase (CES) for HepG2 tumor-dual targeted imaging has not been reported.

**Methods**: Using CBT-Cys click condensation reaction, we rationally designed a “smart” NIR fluorescence probe H_2_N-Cys(StBu)-Lys(Biotin)-Ser(Cy5.5)-CBT (**NIR-CBT**) and used it to facilely prepare the fluorescence-quenched nanoparticle **NIR-CBT-NP**.

**Results**: *In vitro* results indicated that, after **NIR-CBT-NP** was incubated with CES for 6 h, its fluorescence was turned “On” by 69 folds. Cell experiments verified that **NIR-CBT-NP** was uptaken by HepG2 cells *via* biotin receptor-assisted endocytosis and its fluorescence was turned “On” by intracellular CES hydrolysis. Moreover, **NIR-CBT-NP** was successfully applied to image both biotin receptor- and CES-overexpressing HepG2 tumors.

**Conclusion**: Fluorescence-quenched nanoparticle **NIR-CBT-NP** was facilely prepared to actively target biotin receptor-overexpressing HepG2 cancer cells and turn the fluorescence “On” by intracellular CES hydrolysis for tumor-dual targeted imaging. We anticipate that our fluorescence “Turn-On” nanoparticle could be applied for liver cancer diagnosis in clinic in the near future.

## Introduction

With the rapid development of imaging devices and probes, tumor imaging is expected to play a revolutionary role in cancer diagnosis 1. Compared to those tumor imaging techniques of computed tomography 2, ultrasonography 3, photoacoustic imaging 4, magnetic resonance imaging 5, nuclear imaging 6, or positron-emission tomography 7, fluorescence imaging is advantageous in lower cost, higher sensitivity, simpler instrumentation, and easier operation 8. Conventional fluorescence imaging is within the visible range (*i.e.*, 400-800 nm), while near-infrared (NIR) fluorescence imaging red-shifted to 700-900 nm. Thus, tissue absorbance and autofluorescence for NIR fluorescence imaging are largely reduced, leading to higher tissue penetration 9-10. In recent years, NIR fluorescence imaging has become as a promising, noninvasive, high-resolution imaging modality for cancer diagnosis 11.

Fluorescence probes usually have four signal-emitting forms: “Always On”, “Turn-Off”, “Turn-On”, and “Ratiometric” 12-15. Among the four forms, fluorescence “Turn-On” probes have the lowest background signals and thus the highest sensitivity 16-17. Compared with small molecular probes, nanoprobes have tens of thousands of fluorophores and thus dramatically magnified “Turn-On” signals 18-20. Moreover, their longer retention time in cells and tumor regions further enhance their imaging performance 21-22. Recently, Liang and coworkers reported two NIR fluorescence nanoprobes which could be cleaved by tumor-overexpressing enzymes to turn “On” the fluorescence and employed them for tumor imaging 23-24. However, the enzyme cleavage sites of these two nanoprobes are located on the skeletons (not the side chains) of the nanoparticles (NPs), leading to relatively low enzymatic cleavage efficiency.

Beside above enzyme-responsive probes and those smart probes responsive to tumor microenvironment stimuli (*e.g.*, pH, pressure, etc) 25-26, there are lots of probes to label tumor cell surface receptors (*e.g.*, biotin-biotin receptor system) 27 or endothelial cell surface receptors of tumor vessels (*e.g.*, Arg-Gly-Asp (RGD)-α_v_β_3_ integrin system) 28 for active tumor-targeting imaging. And nanoprobes for passive tumor-targeting imaging *via* tumor's enhanced permeability and retention (EPR) effect are more 10. Nevertheless, targeting ability of these single targeting probes is likely impacted by the heterogeneous expression of the receptors or the complicated microenvironment of tumors 29. To solve this problem, modifying the probes with more warheads to target two or more tumor biomarkers might bring about preciser tumor imaging. For example, dual targeting probes could deliver relatively more signal molecules to the tumor site and thus tumor imaging is enhanced 30. Previous studies indicated that both biotin receptor and carboxylesterase (CES) 31 are overexpressed in HepG2 liver cancer cells 32-35. But to the best of our knowledge, simultaneously targeting both biotin receptor and CES for enhanced HepG2 tumor imaging has not been reported.

**Figure 1:**
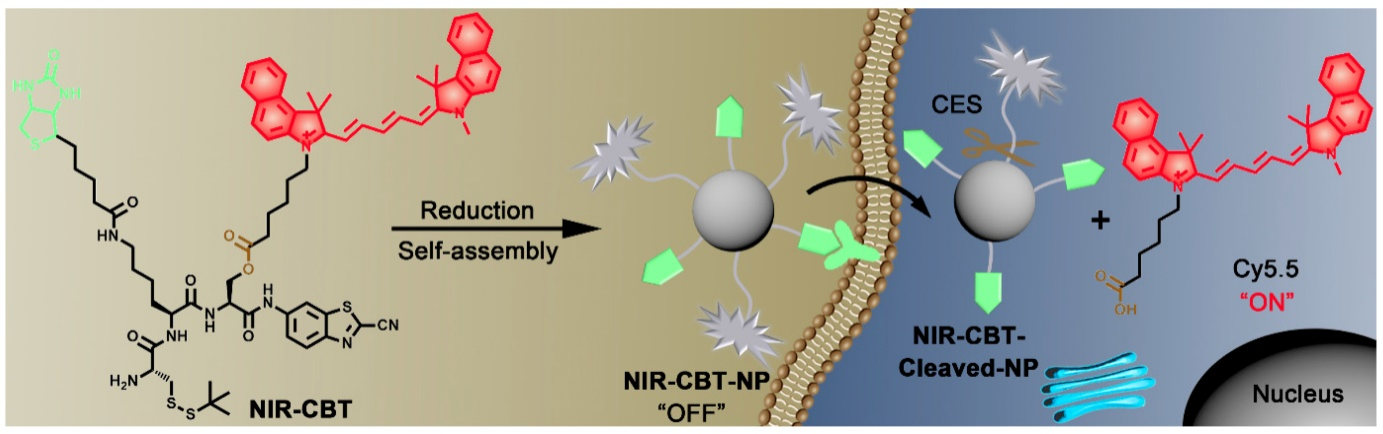
Schematic illustrations of CES-cleavable biotinylated nanoparticle for tumor-dual targeted imaging. NIR probe **NIR-CBT** is subjected to reduction-controlled condensation and self-asssembly to form biotinylated, fluorescence-quenched nanoparticle **NIR-CBT-NP**. **NIR-CBT-NP** targets and translocates the biotin receptor-overexpressing tumor cells and its fluorophore Cy5.5 is subsequently cleaved by intracellular CES, turning the NIR fluorescence signal “On”.

Based on above literature research, herein, we rationally designed a “smart” NIR fluorescence probe H_2_N-Cys(StBu)-Lys(Biotin)-Ser(Cy5.5)-CBT (**NIR-CBT**) and used it to facilely fabricate the fluorescence-quenched nanoparticle **NIR-CBT-NP**, whose fluorescence is turned “On” in HepG2 cancer cells for dual targeting HepG2 tumor imaging. As shown in Figure 1, **NIR-CBT** was designed to contain three components: (1) a 2-cyano-6-aminobenzothiazole (CBT) motif and a latent cysteine (Cys) group for CBT-Cys condensation 36 and subsequent self-assembly to form **NIR-CBT-NP**; (2) a biotin group to target biotin receptor-overexpressing tumor cells; (3) a Cy5.5 fluorophore linked to the side chain of serine (Ser) residue *via* an ester bond to provide the NIR fluoescence and CES cleavage site. Once the disulfide bond of **NIR-CBT** is reduced by a reducing agent (*e.g.*, tris(2-carboxyethyl) phosphine, TCEP), a CBT-Cys click condensation reaction is initiated to self-assemble **NIR-CBT-NP**, accompanied by self-quenching of the Cy5.5 NIR fluorescence. This surface-biotinylated **NIR-CBT-NP** can specifically target biotin receptor-overexpressing tumor cells. After **NIR-CBT-NP** translocating the tumor cells, the ester bonds on their side chains are cleaved by intracellular abundant CES, turning the NIR fluorescence “On”. Since **NIR-CBT-NP** is designed to target biotin receptor and for CES cleavage, only these two biomarker-overexpressing tumors (*e.g.*, HepG2 tumors) are specifically targeted and precisely imaged. Compared with other dual targeting nanoparticles reported 37, our **NIR-CBT**-**NP** nanoparticle was *in vitro* obtained after a covalent condensation reaction which carries following two merits: 1) the synthetic process is very facile; 2) two targeting warheads on the side chains of **NIR-CBT** are readily switched to others by not affecting the condensation reaction. Cell and animal experiments in this work showed that the dual targeted HepG2 cells (or tumors) have the strongest NIR fluorescence.

## Materials and Methods

### Materials and Instruments

CBT was purchased from Shanghai Chemical Pharm-Intermediate Tech. Co.. Electrospray ionization-mass spectrometry (ESI-MS) spectra were obtained on a Q Exactive Plus (ThermoFisher Corporation). ^1^H NMR and ^13^C NMR spectra of **NIR-CBT** were recorded on a Bruker AV 400 MHz spectrometer. High resolution electrospray ionization-mass spectrometry (HR-ESI-MS) spectra of **B**, **C**, **D**, and **NIR-CBT** were recorded on a Finnigan LCQ Advantage ion trap mass spectrometer (ThermoFisher Corporation) which was equipped with a standard ESI source. Dynamic light scattering (DLS) spectrum of **NIR-CBT-NP** was obtained on a NanoBrook 90PLUS PALS particle size analyzer. The spectrum of matrix-assisted laser desorption ionization mass spectrometry (MALDI-MS) was obtained on an Utralextrem III (Bruker Daltonics). An Agilent 1200 HPLC system equipped with a G1322A pump and in-line diode array UV detector was used to conduct high-performance liquid chromatography (HPLC) analyses. An Agilent Zorbax 300SB-C18 RP column, together with CH_3_CN and water (both containing 0.1% trifluoroacetic acid (TFA)) as the eluent, was used for HPLC analysis.

### Preparation of H_2_N-Cys(StBu)-Lys(Biotin)-Ser(Cy5.5)-CBT (NIR-CBT)

**Figure 2:**
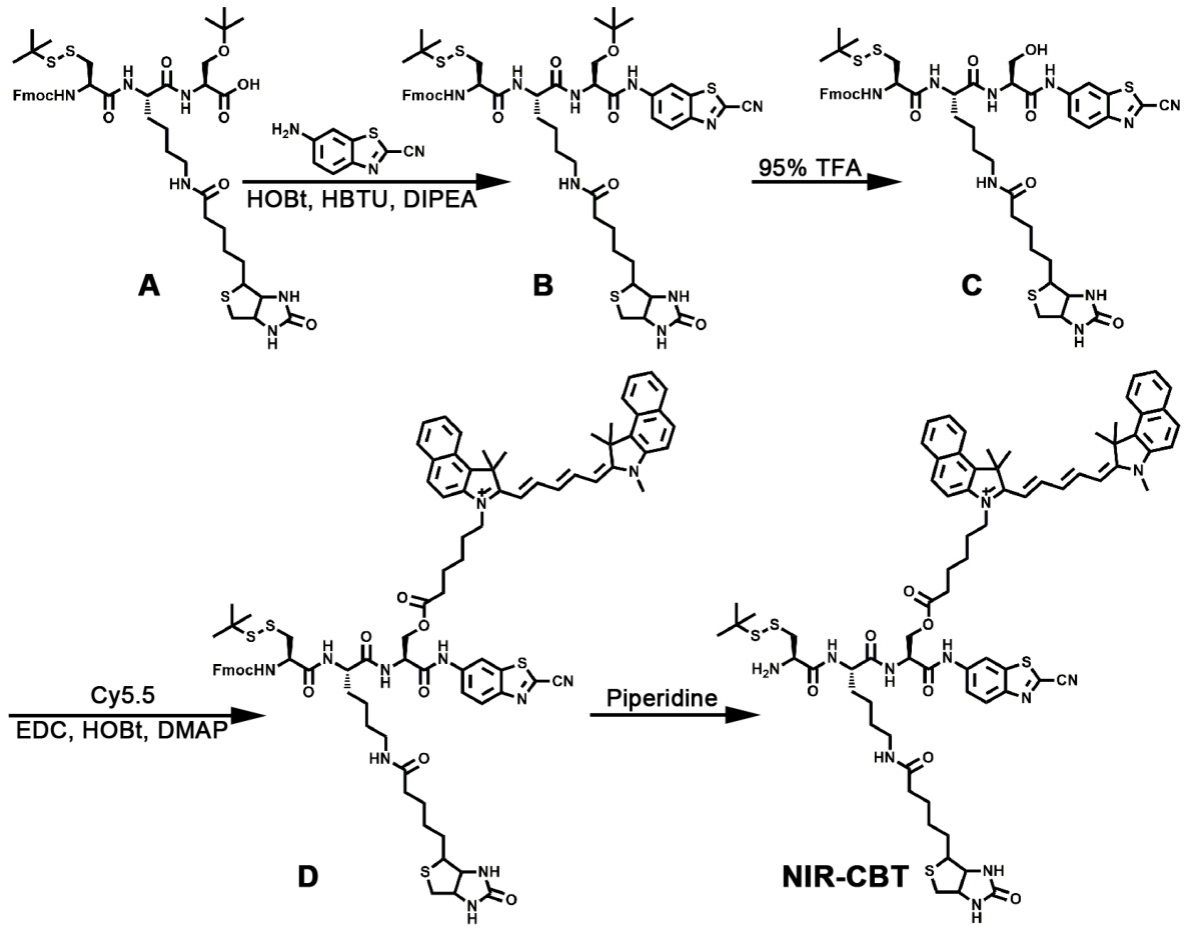
Synthetic route for** NIR-CBT**.

The synthetic route for** NIR-CBT** was illustrated in Figure 2, which consisting of the following 4 steps.

Step 1, synthesis of Fmoc-Cys(StBu)-Lys(Biotin)-Ser(tBu)-CBT (**B**). Compound Fmoc-Cys(StBu)-Lys(Biotin)-Ser(tBu)-OH (**A**) was synthesized through solid phase peptide synthesis. MS: calculated for C_45_H_64_N_6_O_9_S_3_ [M + H]^ +^ = 929.40, obsvd. ESI-MS: *m*/*z* 929.18 (Figure S1). CBT (42 mg, 0.24 mmol) was added to the mixture of **A** (186 mg, 0.2 mmol), 1-hydroxybenzotriazole (HOBt, 32.4 mg, 0.24 mmol), 2-(1H-benzotriazol-1-yl)-1,1,3,3-tetramethyluronium hexafluorophosphate (HBTU, 91 mg, 0.24 mmol) and N,N-diisopropylethylamine (DIPEA, 140 μL, 0.8 mmol) in N,N-dimethylformamide (DMF). Then, the mixture was stirred overnight at room temperature. HPLC was employed for purifying to obtain the pure compound **B** (Table S1). MS: calculated for C_53_H_67_N_9_O_8_S_4_ [M + H]^+^ = 1086.40737, obsvd. ESI-MS: *m*/*z* 1086.40649 (Figure S2).

Step 2, synthesis of Fmoc-Cys(StBu)-Lys(Biotin)-Ser-CBT (**C**). The tBu protecting group of **B** was cleaved with dichloromethane (DCM, 300 μL) and triisopropylsilane (TIPS, 200 μL) in TFA(9.5 mL) at room temperature for 3 h. HPLC was employed to purify compound **C** (Table S1). MS: calculated for C_49_H_59_N_9_O_8_S_4_ [M + H]^+^ = 1030.34477, obsvd. ESI-MS: *m*/*z* 1030.34351 (Figure S3).

Step 3, synthesis of Fmoc-Cys(StBu)-Lys(Biotin)-Ser(Cy5.5)-CBT (**D**). The compound **D** was synthesized through a one-pot reaction. Briefly, **C** (12.4 mg, 0.012 mmol), Cy5.5 (7.2 mg, 0.01 mmol), 1-(3-dimethylaminopropyl)-3-ethylcarbodiimide hydrochloride (EDCŸHCl, 4.8 mg, 0.025 mmol), HOBt (3.4 mg, 0.025 mmol) and 4-dimethylaminopyridine (DMAP, 3.1 mg, 0.025 mmol) were dissolved in DMF and stirred at room temperature overnight. HPLC was employed to purify compound **D** (Table S1). MS: calculated for C_89_H_100_N_11_O_9_S_4_^+^ [M]^+^ = 1594.65828, obsvd. ESI-MS: *m*/*z* 1594.65259 (Figure S4).

Step 4, synthesis of H_2_N-Cys(StBu)-Lys(Biotin)-Ser(Cy5.5)-CBT (**NIR-CBT**). The Fmoc protecting group of **D** was removed with 10% piperidine in DMF (200 μL) at 0 ℃ for 5 min. Then 25 μL TFA was added into the reaction solution to neutralize the alkaline. HPLC was employed to purify the pure final product **NIR-CBT** (Table S1). ^1^H NMR of **NIR-CBT** (400 MHz, DMSO-*d*_6_) δ (ppm): 10.76 (d, *J* = 22.1 Hz, 1 H), 10.76 (d, *J* = 22.1 Hz, 1 H), 8.78 (dd, *J* = 12.8, 8.1 Hz, 1 H), 8.74 - 8.58 (m, 2 H), 8.45 (d, *J* = 6.7 Hz, 1 H), 8.45 - 8.41 (m, 1 H), 8.26 (dd, *J* = 8.3, 5.5 Hz, 2 H), 8.16 (dd, *J* = 9.0, 2.5 Hz, 1 H), 8.06 (dt, *J* = 8.9, 6.9 Hz, 4 H), 8.04 (d, *J* = 4.6 Hz, 1 H), 7.84 - 7.79 (m, 1 H), 7.75 (t, *J* = 7.6 Hz, 2 H), 7.69 (t, *J* = 7.6 Hz, 2 H), 7.66 - 7.59 (m, 1 H), 7.52 (td, *J* = 7.5, 3.4 Hz, 2 H), 6.58 (td, *J* = 12.3, 2.9 Hz, 1 H), 6.30 (ddd, *J* = 33.3, 23.8, 19.5 Hz, 4 H), 4.86 (dd, *J* = 14.1, 6.3 Hz, 1 H), 4.75 (dd, *J* = 13.2, 6.4 Hz, 1 H), 4.53 - 4.40 (m, 1 H), 4.41 - 4.17 (m, 4 H), 4.09 (dt, *J* = 33.7, 17.0 Hz, 4 H), 3.74 (s, 3 H), 3.21 - 2.57 (m, 7 H), 2.59 - 2.52 (m, 9 H), 2.30 (t, *J* = 7.2 Hz, 2 H), 2.10 - 1.99 (m, 4 H), 1.96 (d, *J* = 2.6 Hz, 12 H), 1.65 - 1.35 (m, 14 H) (Figure S5). ^13^C NMR of **NIR-CBT** (100 MHz, DMSO-*d*_6_) δ (ppm): 179.62, 178.53, 177.50, 177.16, 176.36, 173.39, 173.38, 173.30, 171.76, 171.67, 167.96, 163.63, 163.33, 158.22, 157.52, 153.05, 153.00, 145.61, 144.82, 140.15, 138.29, 136.57, 136.44, 135.17, 134.87, 132.97, 132.82, 132.74, 132.43, 130.07, 129.87, 127.40, 127.35, 126.09, 126.00, 119.96, 118.68, 116.85, 116.60, 108.44, 107.78, 66.25, 64.42, 60.65, 55.93, 55.84, 53.22, 53.14, 48.40, 46.37, 43.50, 40.40, 38.55, 36.73, 36.51, 34.56, 34.46, 34.25, 34.20, 33.92, 33.80, 33.47, 33.26, 32.00, 31.84, 31.77, 30.70, 30.55, 30.37, 30.34, 29.27, 27.69, 27.32, 19.17 (Figure S6). MS: calculated for C_74_H_90_N_11_O_7_S_4_^+^ [M]^+^ = 1372.59020, obsvd. ESI-MS: *m*/*z* 1372.58775 (Figure S7).

### Photostability Tests

**NIR-CBT-NP** or Cy5.5 in 10% DMSO-containing PBS was irradiated under 660 nm laser (Beijing Haite Optoelectronic Co., Ltd.) at 0.25 W/cm^2^ for 10 min, respectively. The absorbance of the each sample was measured at various time using UV-Vis analysis (PerkinElmer Lambda 25 UV-vis spectrometer).

### Cell Culture

HepG2 liver cancer cells and LO2 liver normal cells were routinely cultured in Dul-becco's modified Eagle's medium (DMEM, HyClone), containing 10% fetal bovine serum, at 5% CO_2_, 37 °C, and humid atmosphere.

### *In Vitro* Fluorescence “Turn-On” Studies

For the CES cleavage studies, 10 µM **NIR-CBT-NP** (containing 10% dimethyl sulfoxide (DMSO)) was incubated with 0.1 nmol/U CES (Sigma-Aldrich) in phosphate buffer saline (PBS, 10 mM) at 37 °C for 6 h. The solutions were sent for fluorescence measurements and HPLC analyses after incubation. Fluorescence spectra were obtained on a Hitachi F-4600 fluorescence spectrophotometer with excitation wavelength set to 685 nm or emission wavelength set to 720 nm. 25 µM **NIR-CBT-NP** (containing 10% DMSO) in PBS and 25 µM **NIR-CBT-NP** (containing 10% DMSO) incubated with 0.1 nmol/U CES in PBS at 37 °C for 6 h were prepared for transmission electron micrograph (TEM) observation. A JEM-2100F field emission transmission electron microscope operated at an acceleration voltage of 120 kV was employed to obtain TEM images. The diameters of the nanoparticles in the TEM imaging were analyzed by Nano Measurer (1.2).

For the HepG2 cell lysate cleavage studies, the cell lysate was prepared according to the manufacturer's instructions. Briefly, The HepG2 cells growing in log phase were collected and resuspended in RIPA Lysis Buffer (Beyotime Institute of Biotechnology) to the final concentration of 2 × 10^7^/mL. Then, the lysate (1 × 10^7^/mL) was incubated with 10 µM **NIR-CBT-NP** (containing 10% DMSO) in PBS at 37 °C for 4 h, immediately. The solutions were sent for other studies after incubation.

For the cell lysate inhibition studies, the HepG2 cell lysate was first incubated with 10 mM CES inhibitor is-*p*-nitrophenyl phosphate (BNPP, Aladdin) or 10 mM serine protease inhibitor 4-(2-aminoethyl)benzenesulfonyl fluoride hydrochloride (AEBSF, Sigma-Aldrich) at 37 °C for 1 h. Then, **NIR-CBT-NP** (10 µM, containing 10% DMSO) was add to the solution and further incubated at 37 °C for 4 h. The solutions were sent for fluorescence measurements after incubation.

### MTT Assay

3-(4,5-dimethylthiazol-2-yl)-2,5-diphenyltetrazolium bromide (MTT) assay was employed to evaluate the cytotoxicity of **NIR-CBT-NP** or 2% DMSO on HepG2 cells and LO2 cells. Cells growing in log phase were seeded into 96-well cell culture plate at 3 × 10^3^/well. The cells were cultured overnight at 37 °C under 5% CO_2_. The culture medium in each well was removed and the solutions of **NIR-CBT-NP** (100 μL/well, containing 2% DMSO) at concentrations of 2.5, 5, 10, or 20 μM in culture medium were added to the wells, respectively. The HepG2 cells and the LO2 cells were incubated for 6 h or 72 h at 37 °C under 5% CO_2_, respectively. The cells without **NIR-CBT-NP** incubation were used as control for the correction of relative growth rates. To evaluation of the cytotoxicity of 2% DMSO, after the HepG2 cells and LO2 cells were seeded into 96-well cell culture plate and cultured overnight, the culture medium in each well was removed and the solutions of 2% DMSO in culture medium were added to the wells, respectively. The HepG2 cells and the LO2 cells were incubated for 6 h, 12 h, 24 h, 48 h, or 72 h at 37 °C under 5% CO_2_, respectively. The cells without DMSO incubation were used as control for the correction of relative growth rates. 10 μL MTT solution (5 mg/mL, dissolved in PBS buffer) was added to each well of the 96-well plate. After 4 h incubation, the solutions in the wells were removed and the formazan in each well was dissolved with 100 μL DMSO. An enzyme-linked immunosorbent assay (ELISA) reader (VARIOSKAN FLASH) was used to detect the absorption of the solution in each well at 490 nm. The viability of cell growth was calculated using the following formula: viability (%) = (mean of absorbance value of treatment group/mean of absorbance value of control) × 100.

### Cell Apoptosis Assay by Flow Cytometry

Cell apoptosis was measured using Annexin V-FITC Apoptosis Detection Kit (Beyotime Institute of Biotechnology). HepG2 cells and LO2 cells were incubated with 20 μM **NIR-CBT-NP** (containing 2% DMSO) for 6 h and 72 h at 37 °C under 5% CO_2_, respectively. Then the cells were stained with annexin V-fluorescein isothiocyanate (FITC)/propidium iodide (PI) according to the manufacturer's instructions and analyzed by flow cytometry (CytoFLEX, Beckman Coulter).

### Cell Imaging

Fluorescence images of HepG2 cells and LO2 cells were recorded on the IX71 fluorescence microscope (Olympus, Japan). HepG2 cells and LO2 cells growing in log phase were seeded into 6-well cell culture plate at 2 × 10^4^/well and cultured overnight at 37 °C under 5% CO_2_. For the time-course cell imaging studies, HepG2 cells were starved for 2 h and then incubated with 20 µM **NIR-CBT-NP** (containing 2% DMSO) or 20 µM free Cy5.5 (containing 2% DMSO) in culture medium. After being incubated at 37 °C for 0 h, 2 h, 4 h, 6 h, 8 h, 10 h or 12 h, the cells were washed with PBS for three times and then observed by fluorescence microscope. For the comparison with normal liver cells studies, LO2 cells and HepG2 cells were starved for 2 h and then incubated with 20 µM **NIR-CBT-NP** (containing 2% DMSO) in culture medium at 37 °C for 6 h, respectively. For the energy inhibition cell experiments, HepG2 cells were starved for 2 h and then incubated with 20 µM **NIR-CBT-NP** (containing 2% DMSO) in culture medium at 4 °C or 37 °C for 6 h. For the CES inhibition and biotin block studies, the HepG2 cells in the experimental group were starved for 2 h and then incubated with 20 µM **NIR-CBT-NP** (containing 2% DMSO) in culture medium at 37 °C for 6 h. And the HepG2 cells in the other three control groups were starved for 2 h and treated with 1 mM biotin, 100 μM CES inhibitor BNPP, or 1 mM biotin and 100 μM BNPP mixture for 1 h at 37 °C prior to incubation with 20 µM **NIR-CBT-NP** (containing 2% DMSO) in culture medium at 37 °C for further 6 h. At the end of the incubation, the nuclei of the cells were stained with Hoechst 33342 (YEASEN) for further 10 min according to the manufacturer's instructions. Finally the cells were washed with PBS for three times and then observed by fluorescence microscope.

### Statements for the Animal Experiments

All the animals received tender care complied with the guidelines outlined in the Guide for the Care and Use of Laboratory Animals. The procedures were approved by the University of Science and Technology of China Animal Care and Use Committee with an affidavit of Approval of Animal Ethical and Welfare number of USTCACUC1801013.

### Animal Tumor Model

BALB/c nude mice (five week old, weighting 19 - 20 g) were used for animal experiments. HepG2 cells (2 × 10^6^ for each mouse) were subcutaneous injected into the left thigh of each mice. Until the tumor volume reached 50 - 80 mm^3^, the mice were used for subsequent tumor imaging experiments.

### Fluorescence Imaging of HepG2 Tumors

For fluorescence imaging of HepG2 tumors in living animals, all mice were anesthetized using isoflurane gas (2% isoflurane in oxygen, 1 L/min) during all injection and imaging procedures. The tumor-bearing mice were randomly divided into four groups (n = 3 for each group). One group of mice were intratumorally injected with **NIR-CBT-NP** at 0.34 mg/kg, one group of mice were intratumorally injected with the mixture of 0.34 mg/kg** NIR-CBT-NP** and 6.1 mg/kg biotin, one group of mice were injected with the mixture of 0.34 mg/kg** NIR-CBT-NP** and 4.2 mg/kg BNPP, and one group of mice were injected with the mixture of 0.34 mg/kg** NIR-CBT-NP**, 6.1 mg/kg biotin, and 4.2 mg/kg BNPP, all in 10% DMSO-containing PBS. Fluorescence signals generated from the tumors in the nude mice were recorded in real-time by an Xenogen IVIS® spectrum system (IVIS Spectrum; Perkin Elmer). Mice were imaged at different time point after the injection with the excitation filter set to 675 nm and the emission filter set to 720 nm. 24 h after the administration, the mice were sacrificed and the major organs and tumors were taken out, then rinsed with PBS thrice. The fluorescence imagings of the organs and tumors were recorded by an Xenogen IVIS® spectrum system.

## Results and Discussion

### CES-Triggered Fluorescence “Turn-On” of NIR-CBT-NP *in Vitro*

**Figure 3:**
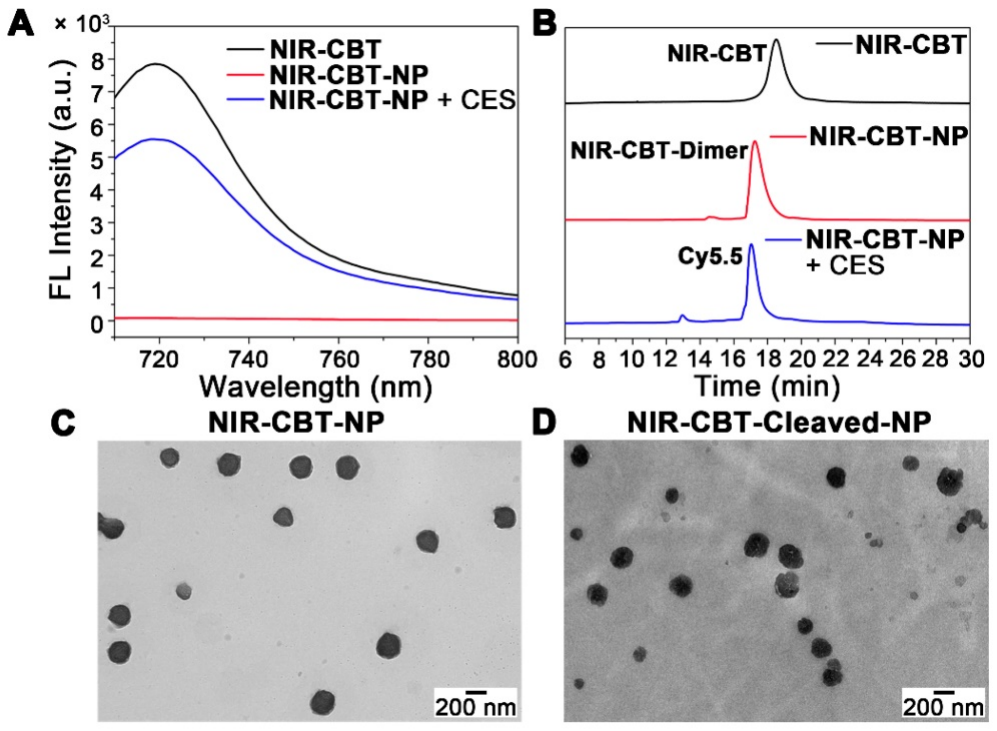
(A) Fluorescence spectra of 10 μM **NIR-CBT** (black), 10 μM **NIR-CBT** incubated with 1 mM TCEP at 37 °C for 1 h (*i.e.*, 10 μM **NIR-CBT-NP** dispersion) (red), and 10 μM **NIR-CBT-NP** incubated with CES (0.1 nmol/U) at 37 °C for 6 h (blue) in PBS. Excitation: 685 nm. (B) HPLC traces of **NIR-CBT** (black), **NIR-CBT-NP** (red), and **NIR-CBT-NP** incubated with CES at 37 °C for 6 h (blue). TEM images of 25 μM **NIR-CBT-NP** dispersion (C) and 25 μM **NIR-CBT-NP** incubated with CES (0.1 nmol/U) at 37 °C for 6 h (D) in PBS. Scale bars, 200 nm.

After synthesis and characterizations of **NIR-CBT** (Figures 2, S1-S7), we used TCEP to trigger the condensation of **NIR-CBT** to self-asssemble **NIR-CBT-NP**. Briefly, 10 μM **NIR-CBT** was incubated with 1 mM TCEP in 40% dimethyl sulfoxide (DMSO)-containing phosphate-buffered saline (PBS, pH 7.4) at 37 °C for 1 h. Then the reaction mixture was centrifuged and the centrifugation was re-dispersed in PBS to yield 10 μM **NIR-CBT-NP**. Dynamic light scattering (DLS) showed the hydrodynamic diameter of the newly prepared **NIR-CBT-NP** was about 339.8 nm (Figure S8). Photostability of **NIR-CBT-NP** was evaluated and compared with that of free Cy5.5. As shown in Figure S9, upon 10 min irradiation of 660 nm laser at 0.25 W/cm^2^, visible absorbance of **NIR-CBT-NP** at 685 nm decreased 59.3% while that of free Cy5.5 decreased 82.0%. These results indicated that formation of **NIR-CBT-NP** effectively improved the photostability of Cy5.5. Fluorescence measurement indicated that, the fluorescence intensity of **NIR-CBT-NP** dispersion at 720 nm dropped to 1/98 of that of **NIR-CBT** (Figure 3A). This fluorescence intensity decrease of **NIR-CBT** was induced by the aggregation-caused quenching (ACQ) effect 38 which herein contributed by two factors: first, formation of **NIR-CBT-Dimer** resulted in the intramolecular quenching of the fluorophore, leading to a ~ 40.2% decrease of the fluorescence intensity; second, self-assembly of **NIR-CBT-Dimer** into **NIR-CBT-NP** in the buffer induced the intermolecular quenching of **NIR-CBT**, which further led to another ~ 59.1% decrease of the fluorescence intensity (Figure S10). When **NIR-CBT-NP** was incubated in PBS for 24 h, the fluorescent change from the solution are negligible (Figure S11), indicating that **NIR-CBT-NP** was stable in the buffer. After incubation of **NIR-CBT-NP** dispersion with CES at 37 °C for 6 h, fluorescence emission of **NIR-CBT-NP** was turned “On” by 69-fold, reaching 70.6% of the original emission of **NIR-CBT** (Figure 3A). High-performance liquid chromatography (HPLC) analyses indicated that, after incubation with TCEP, the peak of **NIR-CBT** disappeared and the peak of its CBT-Cys condensation product (*i.e.*, **NIR-CBT-Dimer** in this work) appeared in **NIR-CBT-NP** solution (Figures 3B, S12). Upon 6 h hydrolysis of **NIR-CBT-NP** by CES, Cy5.5 was completely cleaved from the nanoparticle (Figures 3B, S13), accounting for above fluorescence “Turn-On”. Transmission electron microscopy (TEM) observation verified the existence of the nanoparticles in the **NIR-CBT-NP** dispersion with an average diameter 216.9 ± 28.7 nm (Figure 3C). Interestingly, after **NIR-CBT-NP** incubated with CES, the nanoparticle in the incubation mixture (*i.e.*, **NIR-CBT-Cleaved-NP**) had a decreased diameter of 131.7 ± 21.5 nm but did not disappear (Figure 3D). This was because the CES-cleavable ester bonds are located at the side chains on the particle surface, but not at the skeletons of the nanoparticle as we previously did 23-24. Similar to the results of CES cleavage, the incubation of **NIR-CBT-NP** in CES-overexpressing HepG2 cell lysate at 37 °C for 4 h also induced the detachment of Cy5.5 from the nanoparticle to turn the fluorescence “On” by 74-fold (Figures S14-S15). However, when HepG2 cell lysate was pretreated with a CES inhibitor bis-*p*-nitrophenyl phosphate (BNPP) 39 at 37 °C for 1 h before incubating with **NIR-CBT-NP**, above fluorescence decreased 86.8% (Figure S16). Meanwhile, when HepG2 cell lysate was pretreated with a serine protease inhibitor 4-(2-aminoethyl)benzenesulfonyl fluoride hydrochloride(AEBSF) 40 and then incubated with **NIR-CBT-NP**, very slight fluorescence decrease was observed from the incubation mixture (Figure S16), suggesting the majority of above fluorescence “Turn-On” was induced by CES while other hydrolases contributed the minority. Since numerous esterases including carboxylesterases, and albumins (which are known to have esterase activity) are present in mouse serum 41, we tested the stability of **NIR-CBT-NP** in mouse serum. The results indicated that, even after 24 h incubation of **NIR-CBT-NP** dispersion in mouse serum at 37 °C, fluorescence emission of the nanoparticle was turned “On” by 28-fold (Figure S17), suggesting the nanoparticle is relatively more stable in serum than in HepG2 cells (or tumors). Because the cell culture medium also contains 10% fetal bovine serum, when **NIR-CBT-NP** was incubated with cell culture medium, fluorescence emission of the nanoparticle was turned “On” by 1.6-fold at 6 h or 6.5-fold at 24 h at 37 °C (Figure S18).

### Fluorescence Imaging of Biotin Receptor- and CES-Overexpressing HepG2 Cells with NIR-CBT-NP

**Figure 4:**
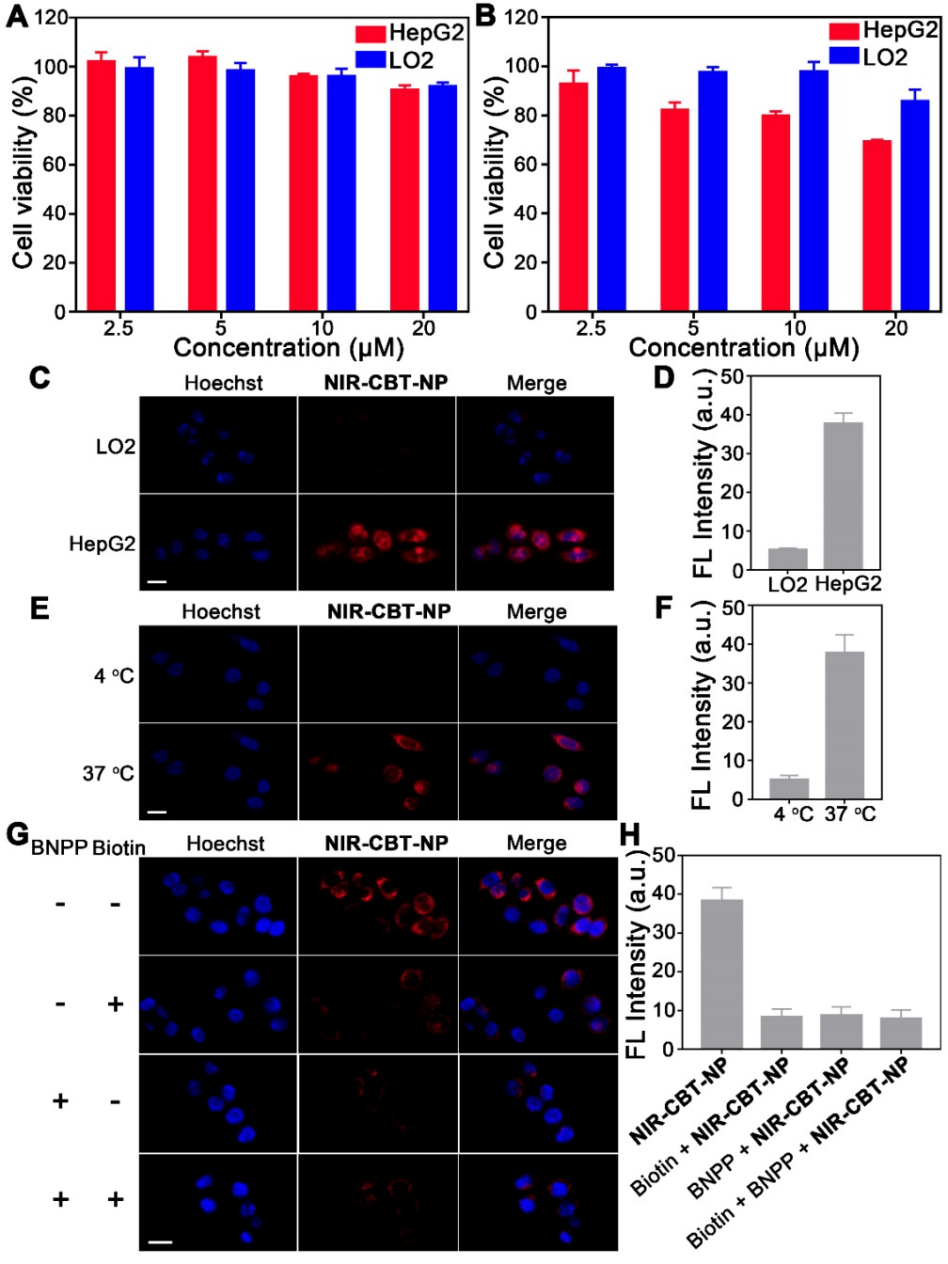
MTT assays of **NIR-CBT-NP** on LO2 cells and HepG2 cells for 6 h (A) and 72 h (B). The experiments were performed in triplicate. Results are representative of three independent experiments. Error bars represent standard deviations. (C) Fluorescence and overlay images of LO2 cells (top row) and HepG2 cells (bottom row) after incubation with 20 μM **NIR-CBT-NP** at 37 °C for 6 h. Scale bar: 20 μm. (D) The mean Cy5.5 fluorescence intensity from LO2 cells or HepG2 cells in C. (E) Fluorescence and overlay images of HepG2 cells after incubation with 20 μM **NIR-CBT-NP** at 4 °C (top row) or 37 °C (bottom row) for 6 h. Scale bar: 20 μm. (F) The mean Cy5.5 fluorescence intensity from HepG2 cells in E. (G) Fluorescence and merged images of biotin receptor-positive and CES-overexpressing HepG2 cells after incubation with 20 μM **NIR-CBT-NP** at 37 °C for 6 h (top row), or pretreated with 1 mM biotin at 37 °C for 1 h then incubated with 20 μM **NIR-CBT-NP** for 6 h (top middle row), or pretreated with 100 μM BNPP for 1 h then incubated with 20 μM **NIR-CBT-NP** for 6 h at 37 °C (bottom middle row), or pretreated with 1 mM biotin and 100 μM BNPP for 1 h then incubated with 20 μM **NIR-CBT-NP** for 6 h at 37 °C (bottom row). Hoechst 33342 (blue), nuclear counterstaining. Red fluorescence, Cy5.5 in **NIR-CBT-NP**. Scale bar, 20 μm. (H) The mean Cy5.5 fluorescence intensity from HepG2 cells in G.

After *in vitro* studies, **NIR-CBT-NP** was subsequently applied for fluorescence imaging of tumor cells. Before that, their cytotoxicity was evaluated on HepG2 liver cancer cells and LO2 liver normal cells using 3-(4,5-dimethylthiazol-2-yl) 2,5 diphenyltetrazplium bromide (MTT) assay. As shown in Figure 4A, up to **NIR-CBT-NP** concentration of 20 µM and incubation time of 6 h, no obvious cytotoxicity was observed from neither HepG2 cells nor LO2 cells, suggesting that 20 µM** NIR-CBT-NP** is safe for live cell imaging. Although 20 µM** NIR-CBT-NP** showed slight cytotoxicity to HepG2 cells after 72 h incubation (69.1% cell viability), no obvious cytotoxicity was observed from the LO2 liver normal cells (Figure 4B). Flow cytometry analysis of 20 µM** NIR-CBT-NP**-treated HepG2 or LO2 cells echoed above MTT results (Figure S19). Meanwhile, 2% DMSO did not show obvious cytotoxicity to neither HepG2 cells nor LO2 cells up to 72 h (Figure S20). We thus chose **NIR-CBT-NP** at 20 µM (containing 2% DMSO) to conduct the following cell imaging study. At 0 h, neglectable background fluorescent signal of 20 µM **NIR-CBT-NP** in culture medium was observed (Figure S21), suggesting very high self-quenching efficiency of **NIR-CBT-NP**. As time went by, fluorescence of the HepG2 cells gradully turned “On”, reaching its intensity plateau at 6 h (increased by 14.7-fold, compared with that at 0 h) then gradually decreased (Figures S21). Fluorescence imaging of the Cy5.5-treated cells indicated that their fluorescence increased to its peak at 2 h and then gradually decreased (Figures S22 and S23). These results indicated that, firstly, **NIR-CBT-NP** could be efficiently taken up by the biotin receptor-overexpressing HepG2 cells and, within 6 h, its fluorescence-quenched Cy5.5 fluorophores could be gradully detached from the nanoparticle upon the hydrolysis of abundant intracellular CES, turning the fluorescence “On”. Secondly, above 1.6-fold background fluorescence “Turn-On” at 6 h in culture medium (Figure S18) would not influence the cell imaging studies (*i.e.*, 14.7-fold fluorescence “Turn-On” at 6 h). We then chose 6 h as the incubation time to conduct the subsequent cell imaging. LO2 liver normal cells were reported low expressing biotin receptors 42. At same incubation condition, Cy5.5 fluorescence of **NIR-CBT-NP** from LO2 cells was only about 13.8% of that from HepG2 cells (Figure 4C&4D), suggesting good selectivity of the nanoparticle for liver cancer cells over liver normal cells. Energy inhibition cell experiments indicated that, fluorescence intensity from cells incubated with **NIR-CBT-NP** at 4 °C was 1/7.4 of that at 37 °C (Figure 4E&4F), suggesting that **NIR-CBT-NP** should be uptaken by HepG2 cells through the biotin receptor-mediated endocytosis. As indicated by the top panels in Figure 4G, after being starved for 2 h and incubated with 20 µM** NIR-CBT-NP** at 37 °C for 6 h, HepG2 cells showed the brightest red NIR Cy5.5 fluorescence from their cytoplasm. To investigate the targeting contribution of the biotin structures on **NIR-CBT-NP** surface to above “Turn-On” fluorescence, HepG2 cells were pretreated with 1 mM biotin for 1 h at 37 °C prior to incubation with the nanoparticle. Cy5.5 fluorescence contrast of the biotin-blocking HepG2 cells was significantly reduced to 21.6% of that of above unblocked cells (top middle row of Figure 4G). To verify above “Turn-On” fluorescence in HepG2 cells was triggered by CES cleavage, we used a CES inhibitor BNPP at 100 μM to pretreat the cells at 37 °C for 1 h before incubated them with **NIR-CBT-NP**. As indicated by the bottom middle row in Figure 4G, the red Cy5.5 fluorescence contrast from the inhibitor-treated cells was significantly reduced to 22.7% of that of the untreated cells, suggesting that it was CES that detached most Cy5.5 from **NIR-CBT-NP** to turn the NIR fluorescence “On”. As expected, those HepG2 cells, whose biotin receptors were blocked and CES activity was inhibited, had the lowest Cy5.5 fluorescence contrast (bottom row in Figure 4G, and Figure 4H). These fluorescence imaging results fully consist with our hypothesis that **NIR-CBT-NP** could actively target and translocate tumor cells overexpressing biotin receptor, followed by intracellular CES hydrolysis to turn “On” the NIR fluorescence.

### Fluorescence Imaging of HepG2 Tumors in Living Animals with NIR-CBT-NP

**Figure 5:**
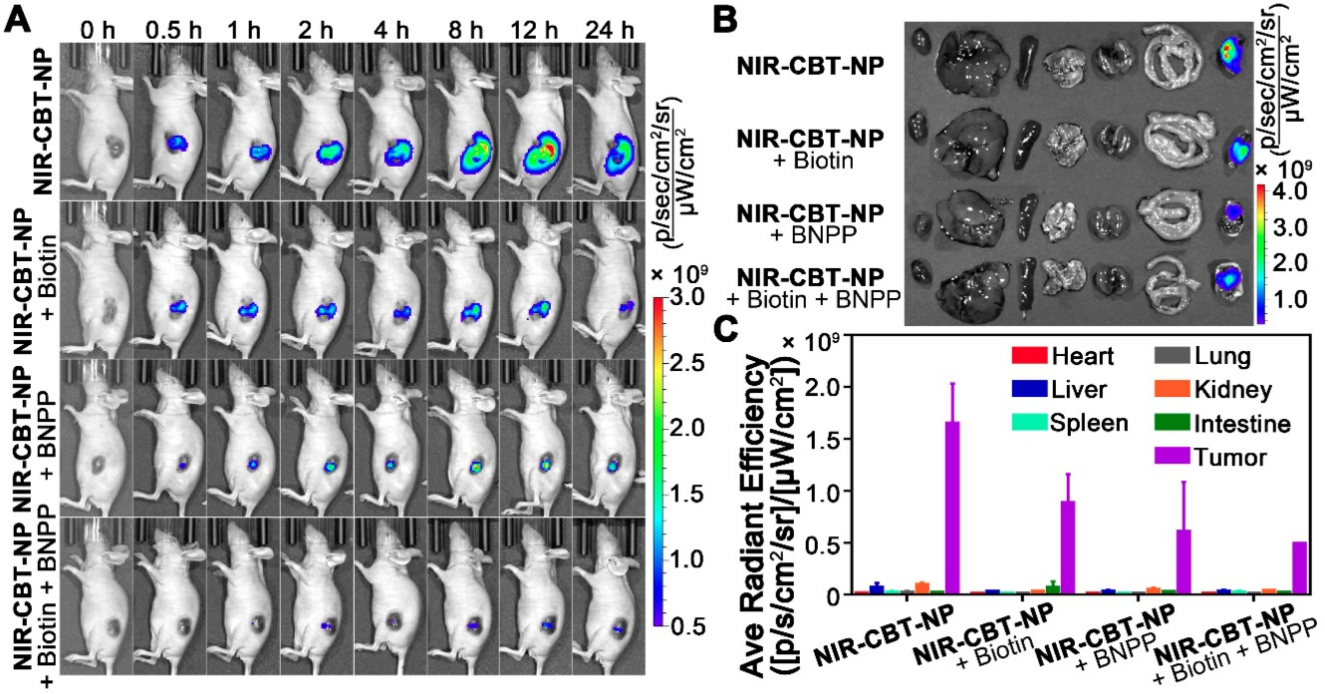
(A) Time-course fluorescence imaging of HepG2 xenograft-bearing nude mice after intratumoral injection of 0.34 mg/kg** NIR-CBT-NP** (top row), 0.34 mg/kg** NIR-CBT-NP** and 6.1 mg/kg biotin (top middle row), 0.34 mg/kg** NIR-CBT-NP** and 4.2 mg/kg BNPP (bottom middle row), or mixture of 0.34 mg/kg **NIR-CBT-NP**, 6.1 mg/kg biotin, and 4.2 mg/kg BNPP (bottom row), in PBS at 0, 0.5, 1, 2, 4, 8, 12, and 24 h. (B) *Ex vivo* fluorescent images of major organs, such as heart, liver, spleen, lung, kidney, intestine and tumor (from left to right) in tumor-bearing mice sacrificed at 24 h after respective intratumoral injections. (C) Quantification of Cy5.5 fluorescence from the major organs (heart, liver, spleen, lung, kidney, intestine and tumor) in B.

We lastly applied **NIR-CBT-NP** for fluorescence imaging of biotin receptor- and CES-overexpressing HepG2 tumors in living animals. The nude mice bearing HepG2 tumor xenografts were randomly divided into four groups (n = 3 for each group). Fluorescence signals generated from the tumors in the nude mice were recorded in real-time by an Xenogen IVIS® spectrum system. For the experimental group, each mouse was intratumorally injected with **NIR-CBT-NP** at 0.34 mg/kg in 10% DMSO-containing PBS (top row in Figure 5A). And for the three control groups, one group of mice were intratumorally injected with the mixture of 0.34 mg/kg** NIR-CBT-NP** and 6.1 mg/kg biotin (top middle row in Figure 5A), one group of mice were injected with the mixture of 0.34 mg/kg** NIR-CBT-NP** and 4.2 mg/kg BNPP (bottom middle row in Figure 5A), and one group of mice were injected with the mixture of 0.34 mg/kg** NIR-CBT-NP**, 6.1 mg/kg biotin, and 4.2 mg/kg BNPP (bottom row in Figure 5A), all in 10% DMSO-containing PBS. As indicated by Figure 5A, the mice in the experimental group had the strongest fluorescent signal from the tumor sites, and the signal increased with time, reaching to its plateau at 12 h then gradually decreased (top row in Figure 5A, Figure S24). The mice in two control groups, one of whose two biomarkers was inhibited (*i.e.*, either biotin receptor was inhibited in the second row or CES was inhibited in the third row), showed relatively weaker fluorescence from the tumor sites (two middle rows in Figure 5A). As expected, the mice whose two biomarkers were both inhibited showed the weakest fluorescence from their tumor sites (bottom row in Figure 5A, Figure S24). These* in vivo* imaging results demonstrated that **NIR-CBT-NP** could target the tumor cells overexpressing biotin receptor and be hydrolyzed by CES inside cells, turning NIR fluorescent signal “On” for tumor-dual targeted imaging. After imaging, the mice were sacrificed and the major organs (heart, liver, spleen, lung, kidney, and intestine) and tumors were taken out for *ex vivo* imaging. As shown in Figure 5B and 5C, strong NIR fluorescent signals were found from the tumors but not from other organs, aggreeing with above *in vivo* tumor imaging results.

## Conclusions

In summary, we facilely prepared the fluorescence-quenched nanoparticle **NIR-CBT-NP** to actively target biotin receptor-overexpressing HepG2 cancer cells and turn the fluorescence “On” by intracellular CES hydrolysis for tumor-dual targeted imaging. *In vitro* results indicated that, after **NIR-CBT-NP** was incubated with CES for 6 h, its fluorescence was turned “On” by 69 folds and its original 217 nm nanoparticle size was reduced to 132 nm. Cell experiments verified that **NIR-CBT-NP** was uptaken by HepG2 cells *via* biotin receptor-assisted endocytosis and its fluorescence was turned “On” by intracellular CES hydrolysis. Moreover, **NIR-CBT-NP** was successfully applied to image biotin receptor- and CES-overexpressing HepG2 tumors. We anticipate that our fluorescence “Turn-On” nanoparticle could be applied for liver cancer diagnosis in clinic in the near future.

## Supplementary Material

Supplementary figures and tables.
