# Supplementary material for: Carboxylesterase-Cleavable Biotinylated Nanoparticle for Tumor-Dual Targeted Imaging
Source: Theranostics. 2019 Sep 25;9(24):7359–69. doi: 10.7150/thno.37625 (PMC6831296; doi:10.7150/thno.37625)
Supplement: Supplementary file 1 — Supplementary figures and tables. [file thnov09p7359s1.pdf]

Supplementary Material

Carboxylesterase-Cleavable Biotinylated Nanoparticle for Tumor-Dual Targeted Imaging

Peiyao Chen,[1] Wen Kuang,[1] Zhen Zheng,[1] Shuye Yang,[2] Yaling Liu,[3] Lanhong Su,[4] Kui Zhao,*[2] and Gaolin Liang*[1]

1. Hefei National Laboratory of Physical Sciences at Microscale, Department of Chemistry, University of Science and Technology of China, 96 Jinzhai Road, Hefei, Anhui 230026, China
2. Department of PET Center, The First Affiliated Hospital, College of Medicine, Zhejiang University, 79 Qingchun Road, Hangzhou, Zhejiang 310003, China
3. Jiangsu Institute of Nuclear Medicine, 20 Qianrong Road, Wuxi, Jiangsu 214063, China
4. School of Life Sciences, University of Science and Technology of China, 443 Huangshan Road, Hefei, Anhui 230027, China

E-mail: zhaokui0905@zju.edu.cn (K. Z.), gliang@ustc.edu.cn (G.-L. L.).

Supplementary figures and table

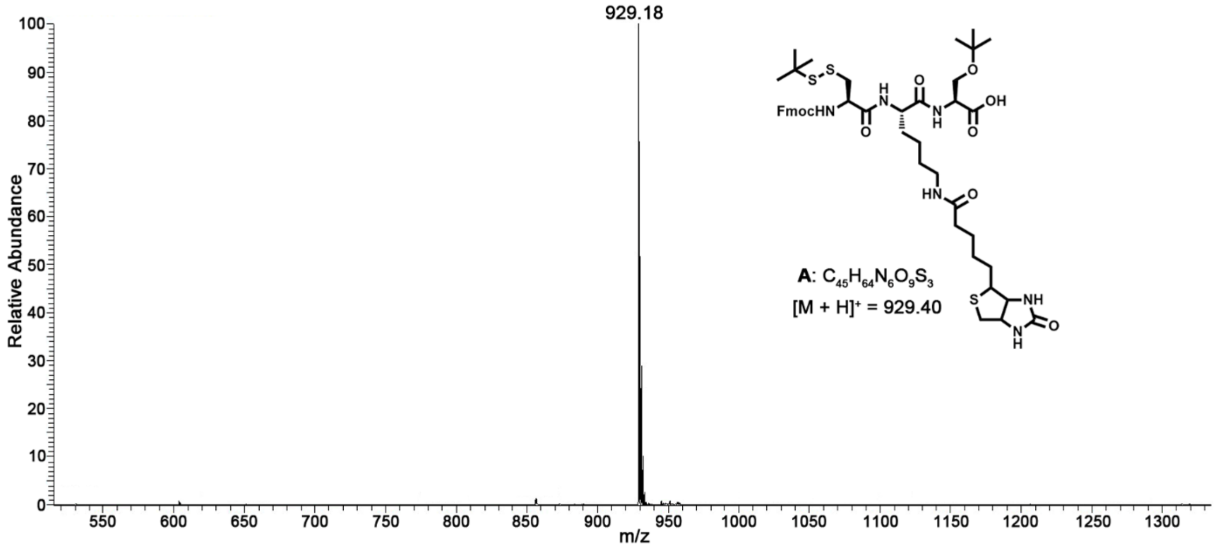

Figure S1. ESI/MS spectrum of **A**.

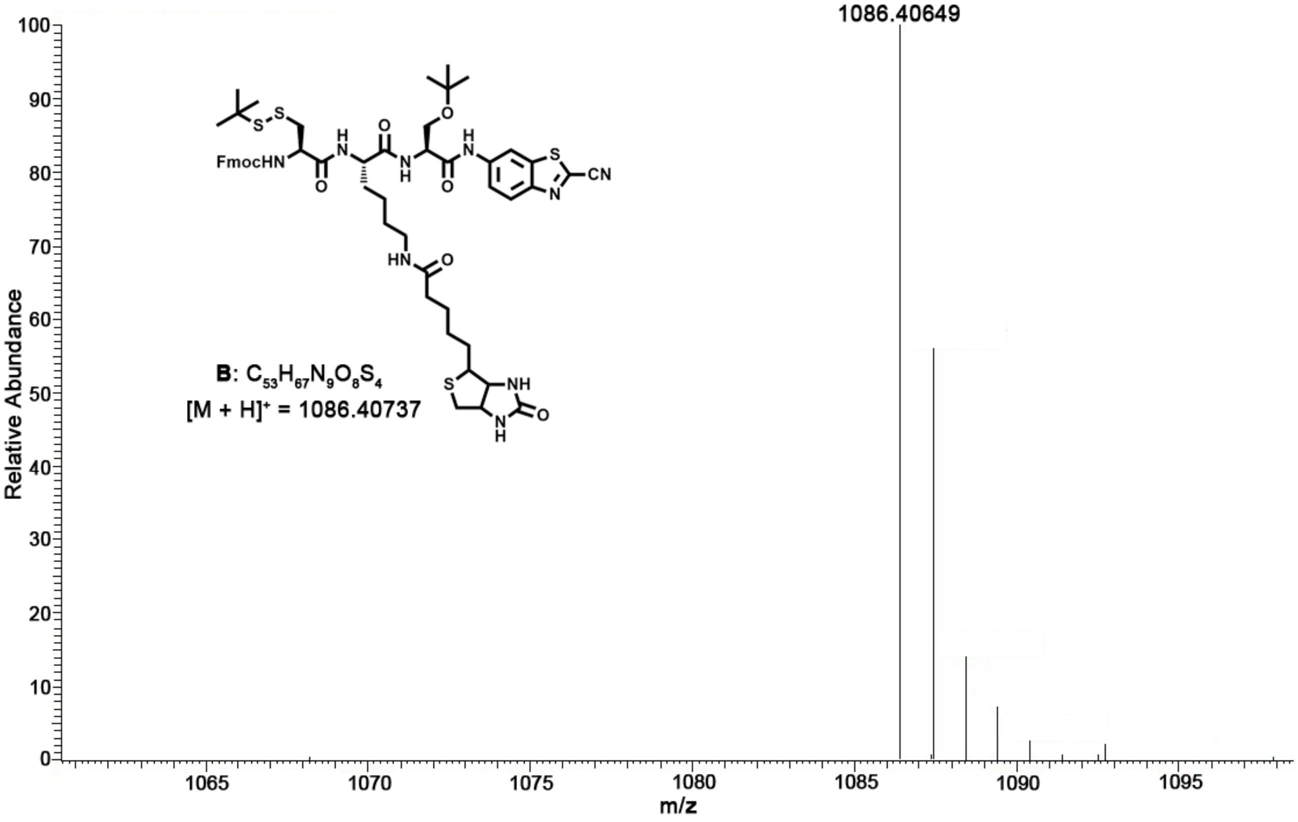

Figure S2. HR-ESI/MS spectrum of **B**.

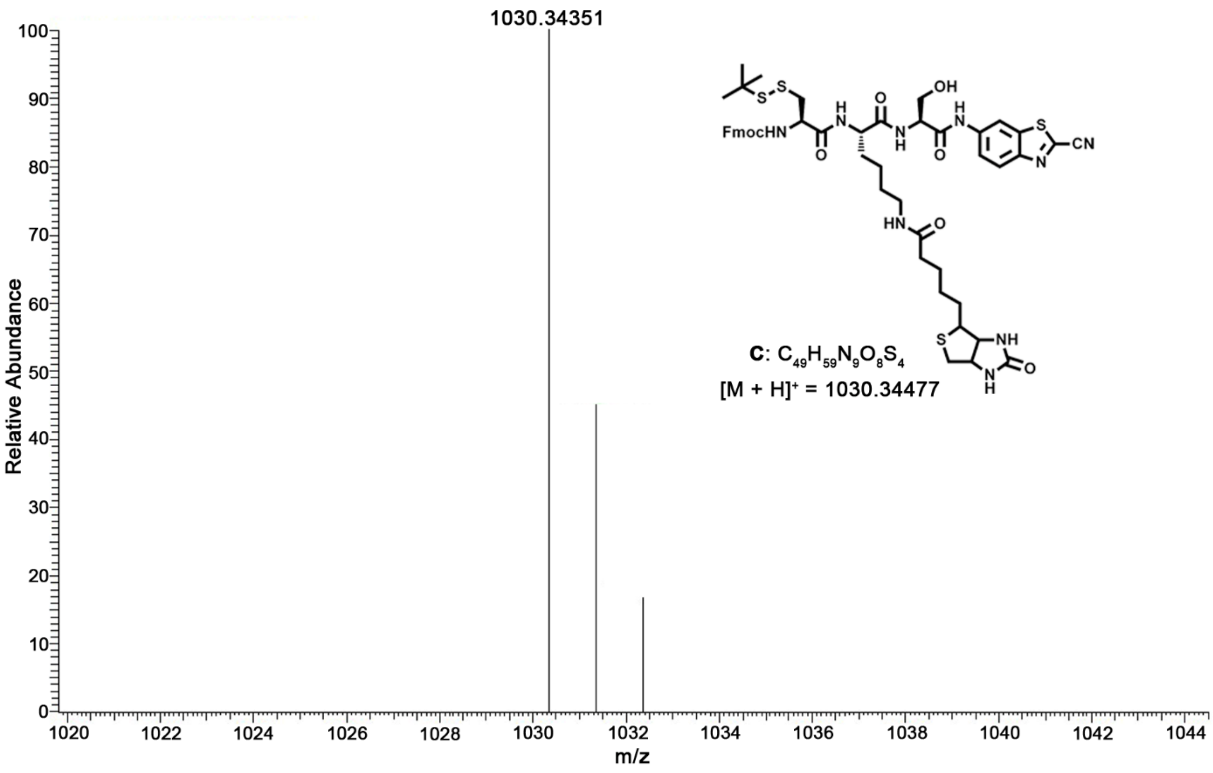

Figure S3. HR-ESI/MS spectrum of **C**.

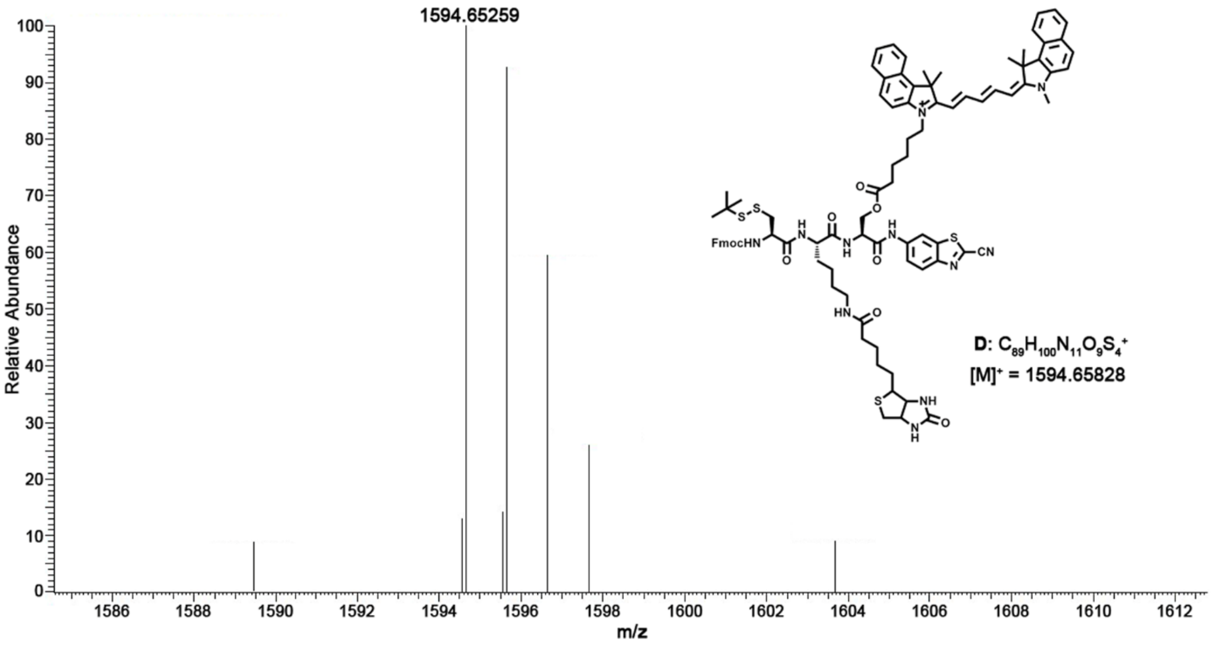

Figure S4. HR-ESI/MS spectrum of **D**.

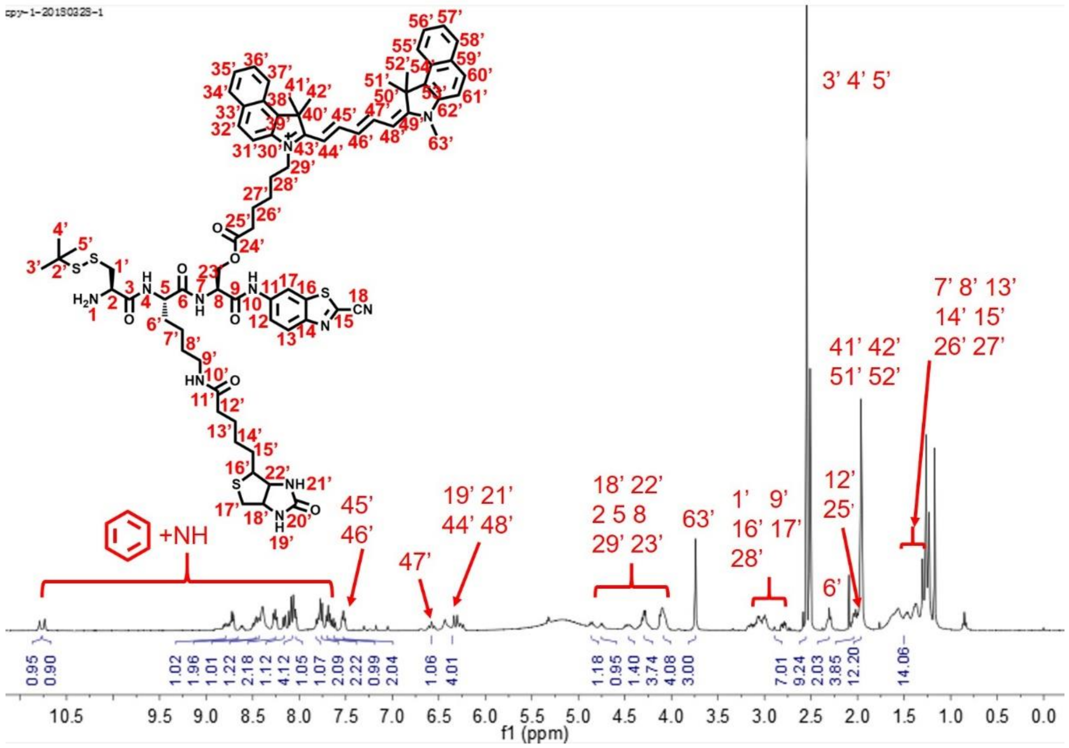

Figure S5. ^{1}H NMR spectrum of **NIR-CBT**.

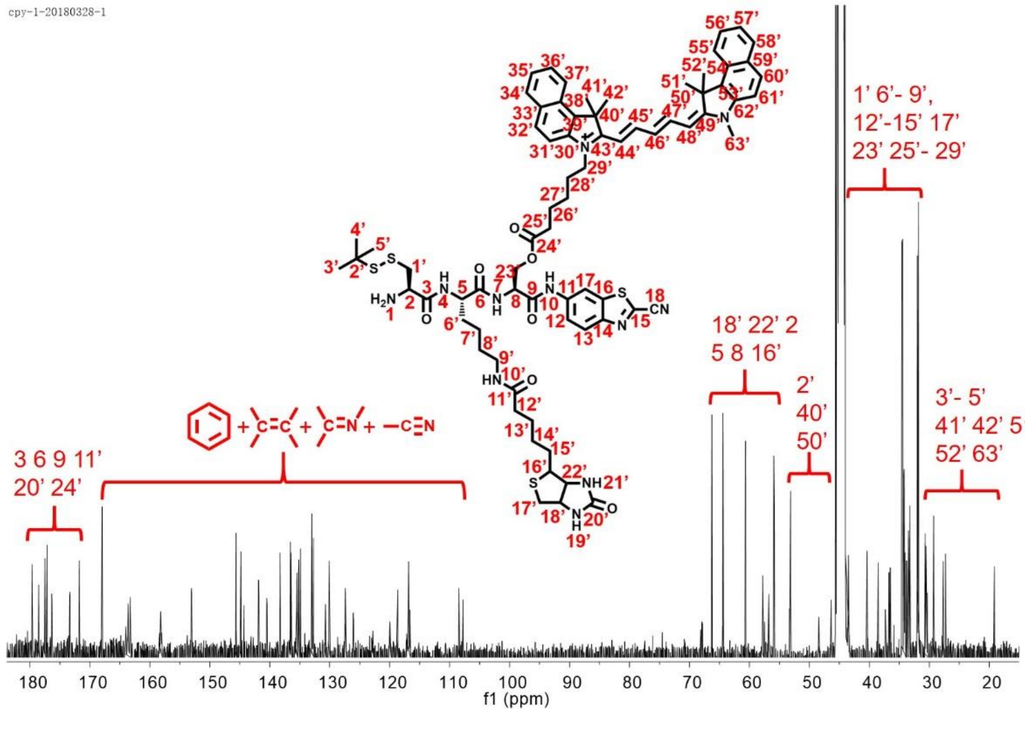

Figure S6. ^{13}C NMR spectrum of **NIR-CBT**.

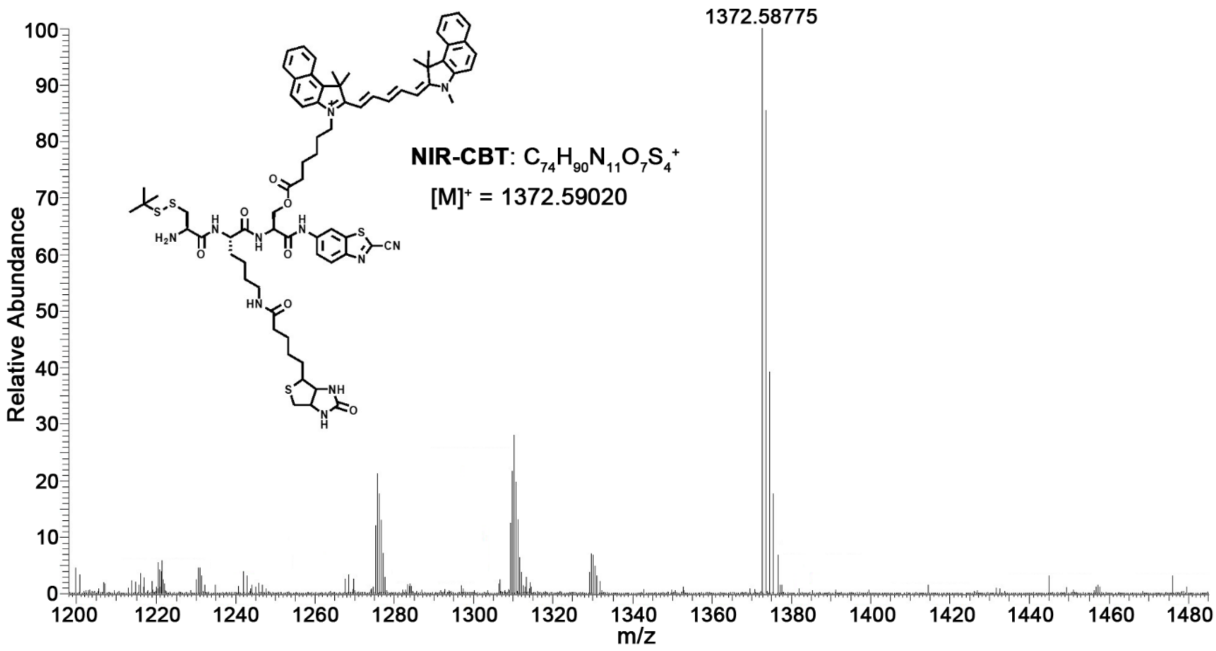

Figure S7. HR-ESI/MS spectrum of **NIR-CBT**.

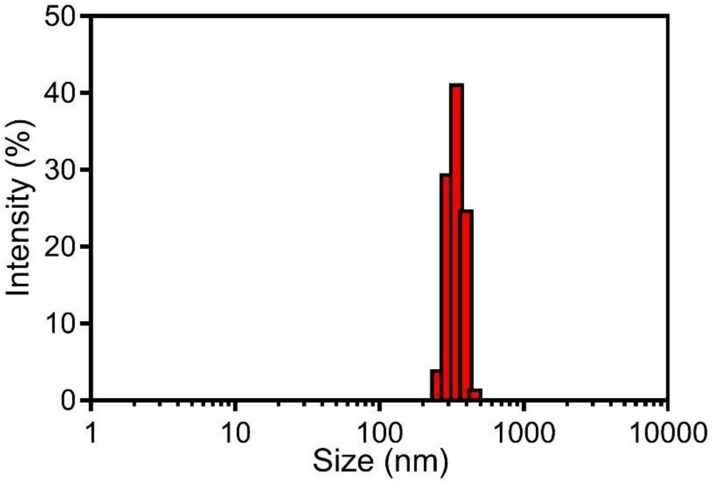

Figure S8. DLS measurement of **NIR-CBT-NP**.

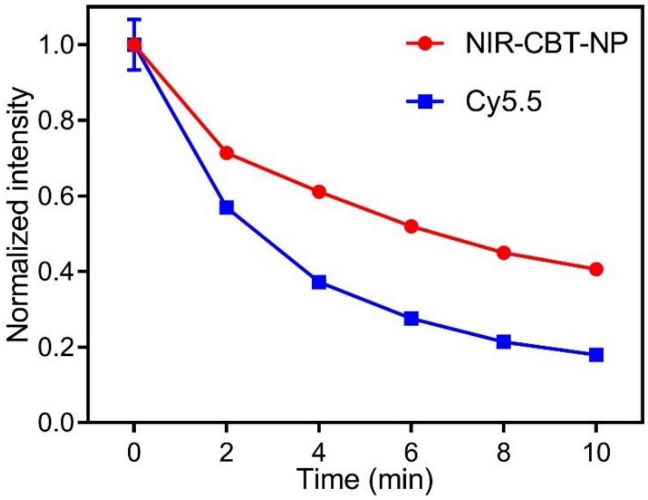

Figure S9. Normalized absorbances at 685 nm of 10 μM **NIR-CBT-NP** or free Cy5.5 in PBS (containing 10% DMSO) under 660 nm irradiation (0.25 W/cm^2) for 0, 2, 4, 6, 8, or 10 min, respectively. Results are presented as mean ± S.D., n = 3.

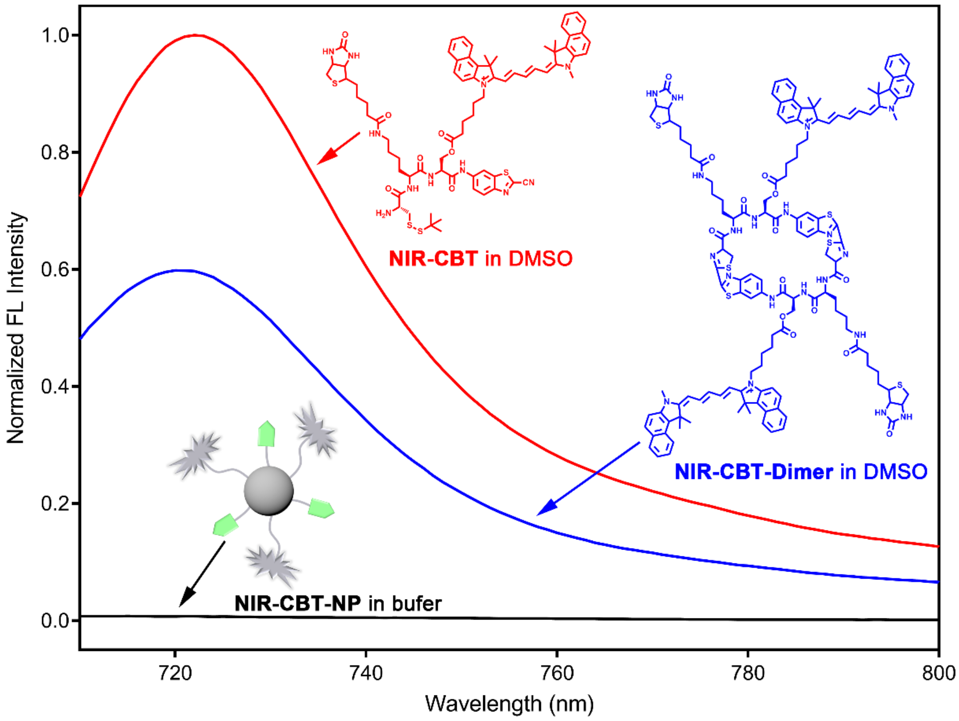

Figure S10. Normalized fluorescence spectra of 10 μM **NIR-CBT** dissolved in DMSO (red), 10 μM **NIR-CBT-NP** dissolved in DMSO (*i.e.*, **NIR-CBT-Dimer**, blue) and 10 μM **NIR-CBT-NP** dissolved in buffer (black).

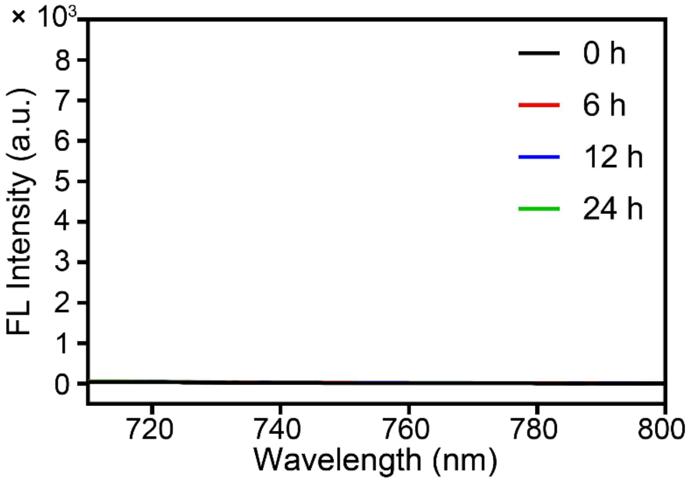

Figure S11. Fluorescence spectra of 10 μM **NIR-CBT-NP** in PBS at 37 ℃ for 0 h (black), 6 h (red), 12 h (blue), or 24 h (green). Excitation: 685 nm.

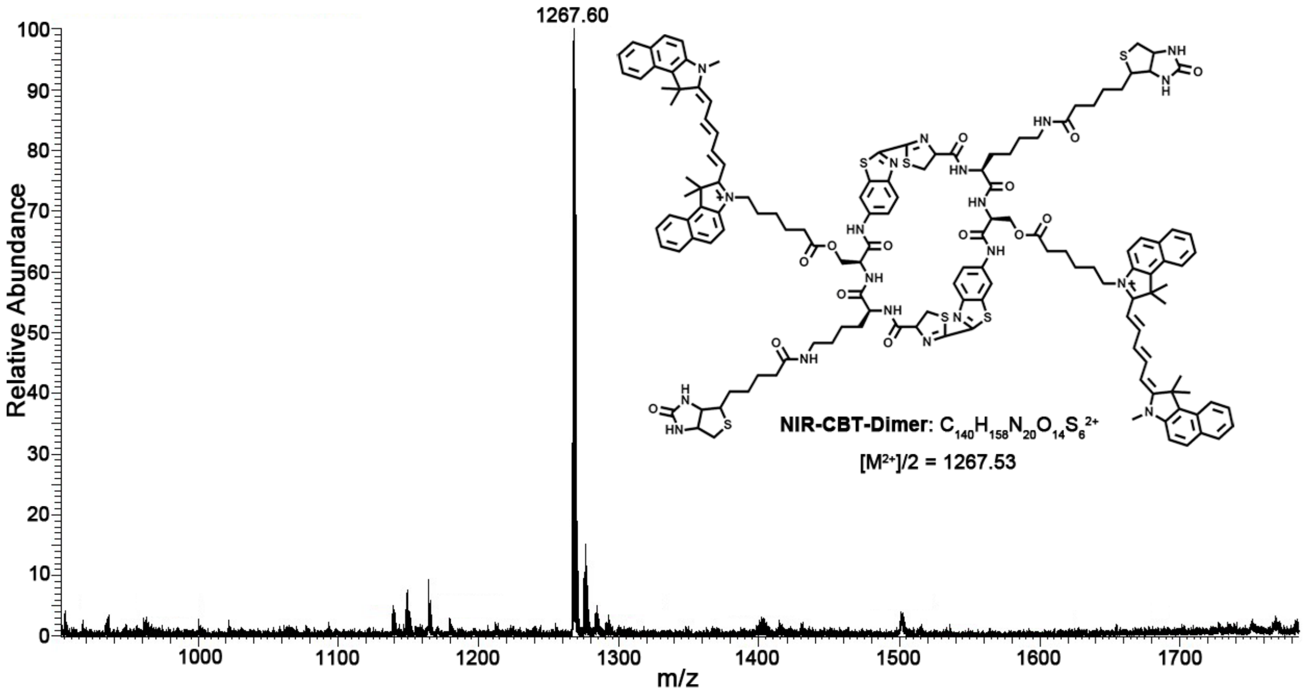

Figure S12. ESI-MS spectrum of HPLC peak at 17.2 min in Figure 3B.

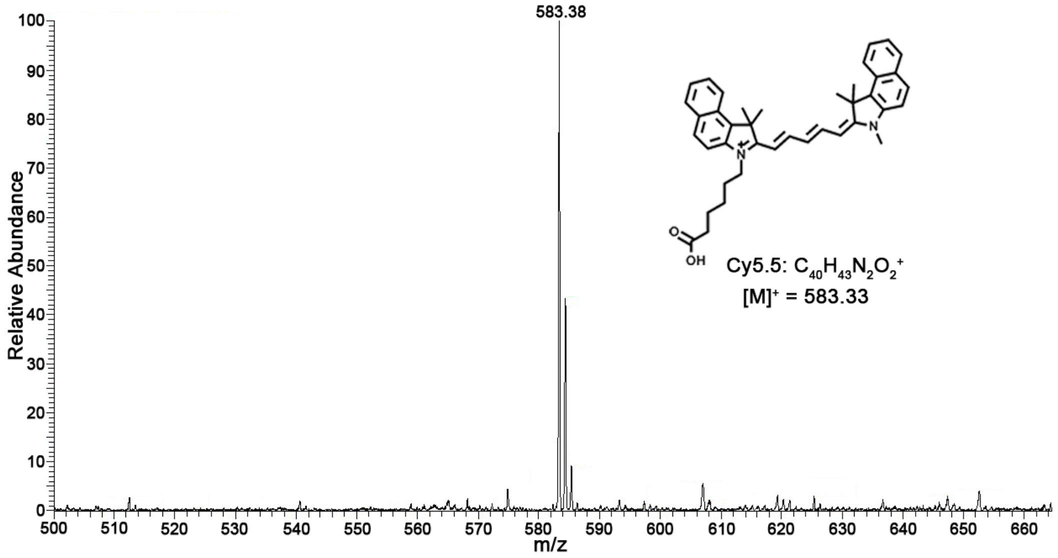

Figure S13. ESI-MS spectrum of HPLC peak at 17.0 min in Figure 3B.

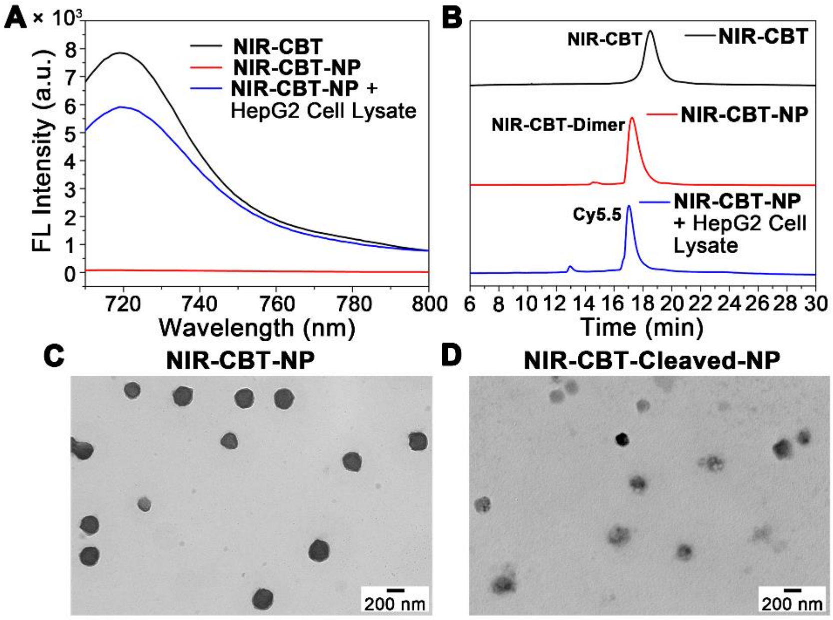

Figure S14. (A) Fluorescence spectra of 10 μM **NIR-CBT** (black), 10 μM **NIR-CBT** incubated with 1 mM TCEP at 37 ℃ for 1 h (*i.e.*, 10 μM **NIR-CBT-NP** dispersion) (red), and 10 μM **NIR-CBT-NP** incubated with HepG2 cell lysate at 37 ℃ for 4 h (blue) in PBS. Excitation: 685 nm. (B) HPLC traces of **NIR-CBT** (black), **NIR-CBT-NP** (red), and **NIR-CBT-NP** incubated with HepG2 cell lysate for at 37 °C for 4 h (blue). TEM images of 25 μM **NIR-CBT-NP** dispersion (C) and 25 μM **NIR-CBT-NP** incubated with HepG2 cell lysate at 37 ℃ for 4 h (D) in PBS. Scale bars, 200 nm.

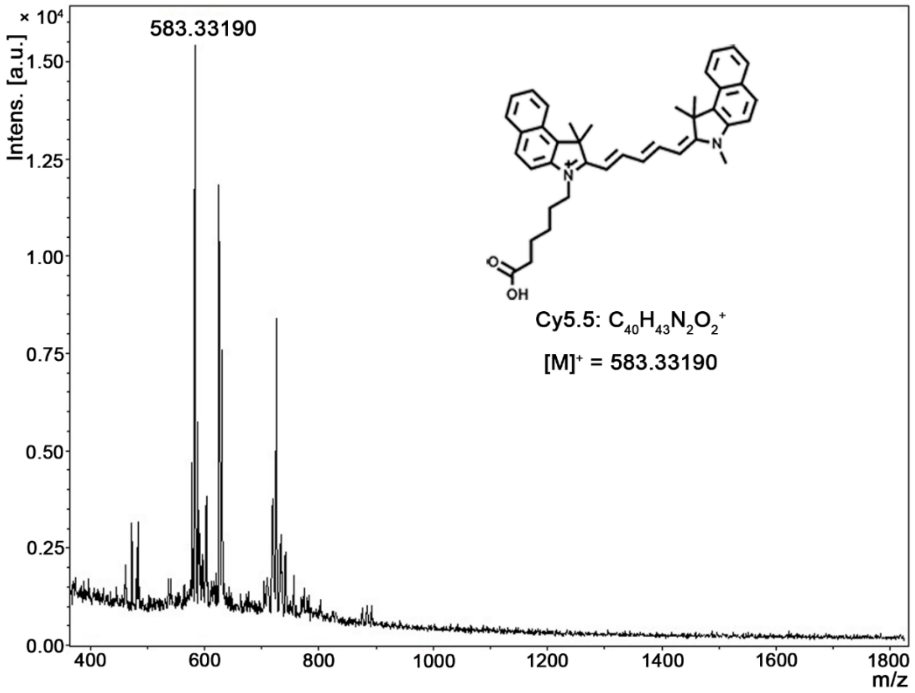

Figure S15. MALDI-MS spectrum of HPLC peak at 17.0 min in Figure S14.

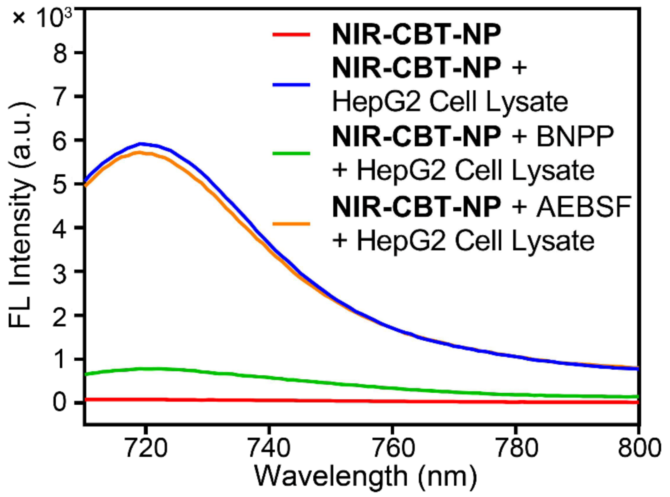

Figure S16. Fluorescence spectra of 10 μM **NIR-CBT-NP** (red), 10 μM **NIR-CBT-NP** incubated with HepG2 cell lysate at 37 ℃ for 4 h (blue) in PBS, 10 μM **NIR-CBT-NP** incubated with CES inhibitor BNPP (10 mM)-pretreated HepG2 cell lysate at 37 ℃ for 4 h (green) in PBS, and 10 μM **NIR-CBT-NP** incubated with serine protease inhibitor AEBSF (10 mM)-pretreated HepG2 cell lysate at 37 ℃ for 4 h (orange) in PBS. For inhibition experiments, HepG2 cell lysate was pretreated with BNPP or AEBSF at 37 ℃ for 1 h, before incubated with **NIR-CBT-NP**, respectively. Excitation: 685 nm.

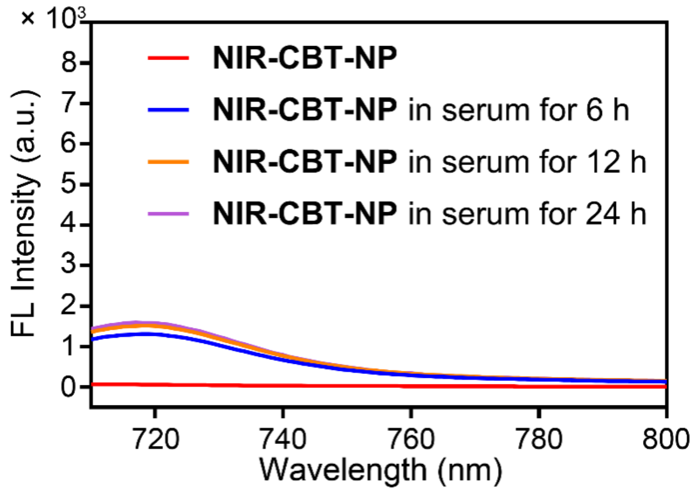

Figure S17. Fluorescence spectra of 10 μM **NIR-CBT-NP** (red), 10 μM **NIR-CBT-NP** incubated in mouse serum at 37 ℃ for 6 h (blue), 12 h (orange), or 24 h (purple). Excitation: 685 nm.

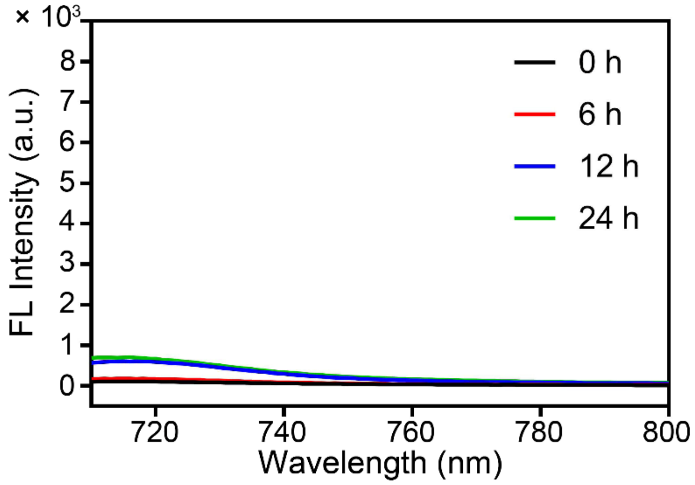

Figure S18. Fluorescence spectra of 10 μM **NIR-CBT-NP** in cell culture medium at 37 ℃ for 0 h (black), 6 h (red), 12 h (blue), or 24 h (green). Excitation: 685 nm.

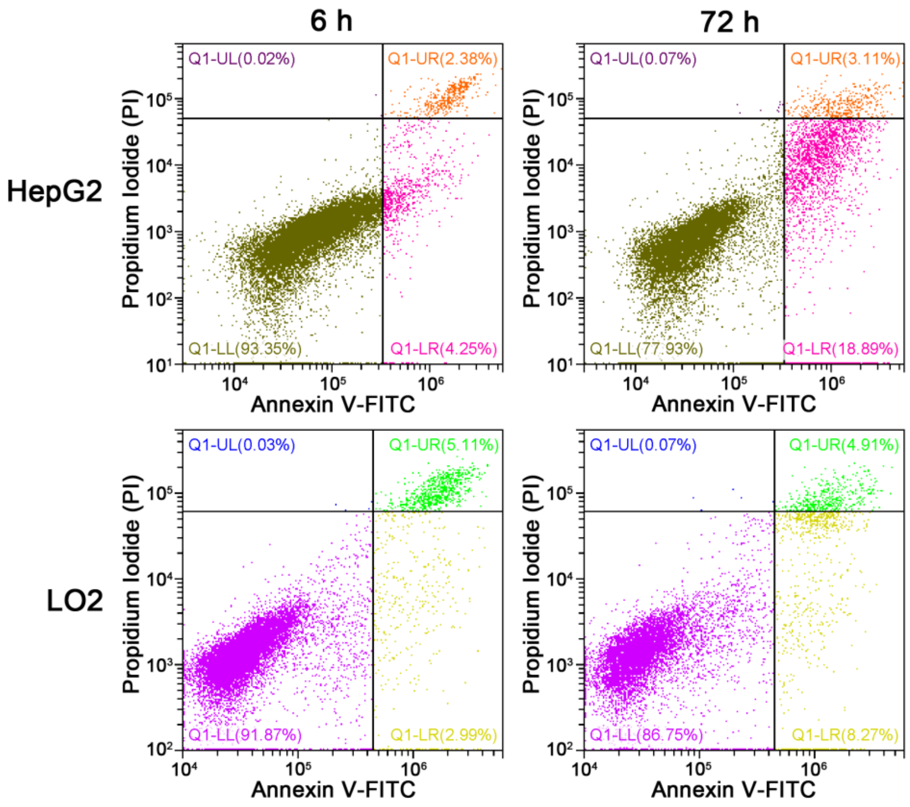

***Figure S19*.** Effects of **NIR-CBT-NP** on HepG2 cells or LO2 cells apoptosis. The cells were treated with 20 μM **NIR-CBT-NP** for 6 h or 72 h, respectively. Apoptosis was evaluated by annexin V-FITC and PI staining followed by flow cytometry analysis. Percentage of necrotic cells (Q1-UL: annexin V-FITC$^-$/PI$^+$), late apoptosis cells (Q1-UR: annexin V-FITC$^+$/PI$^+$), early apoptosis cells (Q1-LR: annexin V-FITC$^+$/PI$^-$), and living cells (Q1-LL: annexin V-FITC$^-$/PI$^-$).

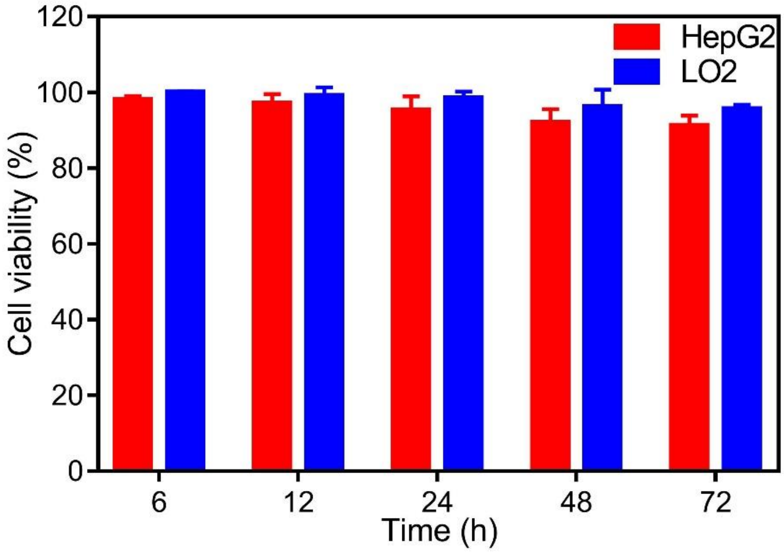

Figure S20. MTT assay of 2% DMSO on HepG2 cells and LO2 cells. The experiments were performed in triplicate. Results are representative of three independent experiments. Error bars represent standard deviations.

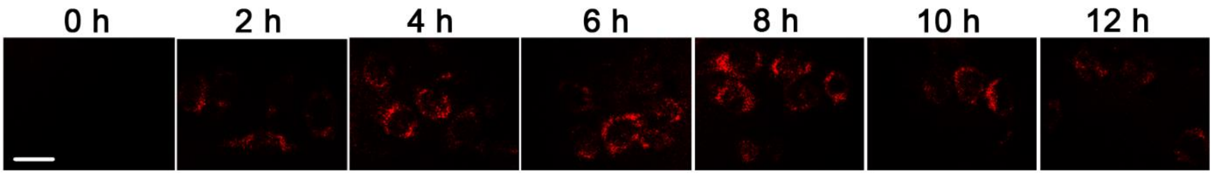

Figure S21. Time course fluorescence-microscopic images of HepG2 cells incubated with 20 μM **NIR-CBT-NP** in culture medium containing 2% DMSO at 37 °C. All images have the same scale bar: 20 μm.

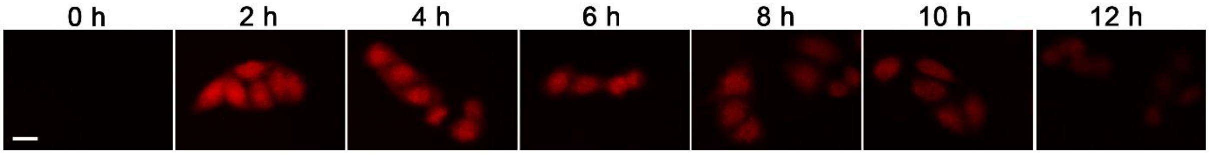

Figure S22. Time course fluorescence-microscopic images of HepG2 cells incubated with 20 μM Cy5.5 in culture medium containing 2% DMSO at 37 °C. All images have the same scale bar: 20 μm.

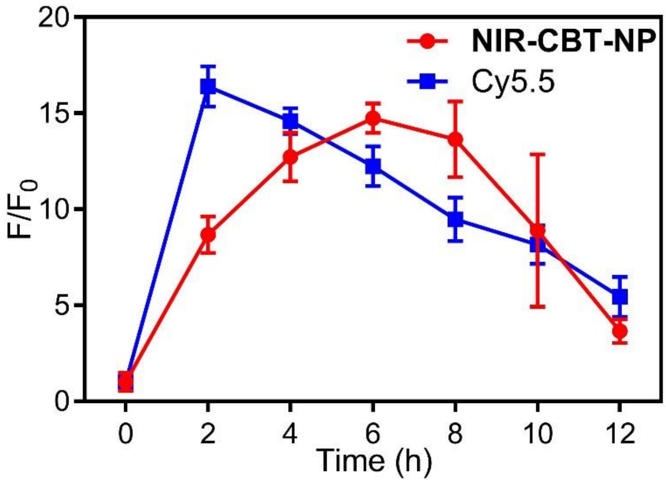

Figure S23. Time course of the mean Cy5.5 fluorescence intensity from HepG2 cells treated with **NIR-CBT-NP** (red) or Cy5.5 (blue) verse that at 0 h in Figure S21 and S22, respectively.

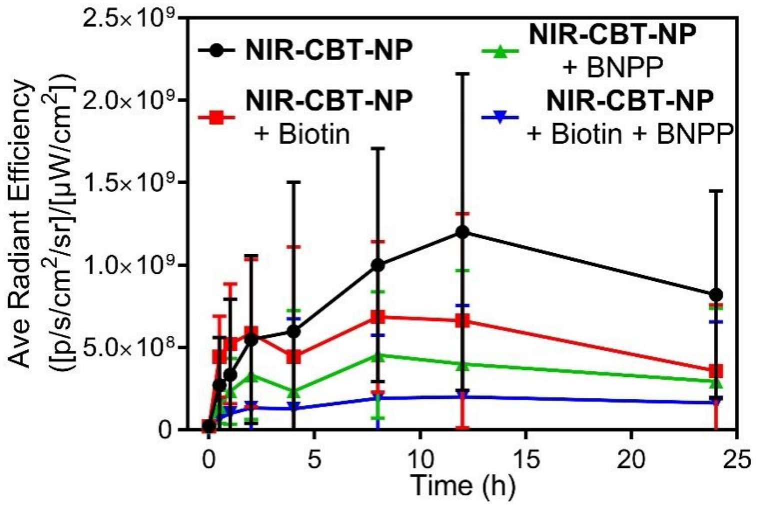

Figure S24. Quantification of the average radiant efficiency ([p/s/cm^2/sr]/[μW/cm^2]) from the tumor regions for the mouse images in Figure 5.

Table S1. HPLC condition for the purification of the compounds in Figure 2, Figures 3B and S14B.

Time (min)	Flow (mL/min)	H_2O % (0.1 % TFA)	CH_3CN % (0.1 % TFA)
0	1.0	50	50
3	1.0	50	50
35	1.0	5	95
37	1.0	5	95
38	1.0	50	50
40	1.0	50	50
